# Fathers’ involvement in raising children with intellectual disabilities: Mothers’ ratings of the contribution of their spouses

**DOI:** 10.1371/journal.pone.0294077

**Published:** 2024-05-22

**Authors:** Ahmed Mohamed, Maxwell Peprah Opoku, Mohammed Safi, Shashidhar Belbase, Fadwa Al Mughairbi, Quizhi Xie, Mahmoud Al Shatheli, Shamsa Almarzooq

**Affiliations:** 1 Department of Special and Gifted Education, United Arab Emirates University, Al Ain, Abu Dhabi, United Arab Emirates; 2 Department of Speech Language Pathology, United Arab Emirates University, Al Ain, Abu Dhabi, United Arab Emirates; 3 Curriculum and Methods of Instruction, United Arab Emirates University, Al Ain, Abu Dhabi, United Arab Emirates; 4 Department of Clinical Psychology, United Arab Emirates University, Al Ain, Abu Dhabi, United Arab Emirates; 5 Zayed Higher Organisation, Al Ain, Abu Dhabi, United Arab Emirates; Jacobi Medical Center: New York City Health and Hospitals Jacobi, UNITED STATES

## Abstract

**Background:**

Intellectual disability (ID) is a lifelong condition characterized by individuals’ inability to perform cognitive tasks and participate in daily living activities. While parenting children with ID has been reported to be demanding, studies draw mainly on mothers. In contexts such as the United Arab Emirates (UAE), there is little literature on fathers’ involvement in raising children with IDs.

**Objectives:**

The purpose of this study was to explore, from the perspectives of mothers, the extent of fathers’ involvement in raising children with ID in the UAE.

**Methods:**

One hundred and fifty-eight (N = 158) mothers with children with ID completed the fathers’ involvement in disability and rehabilitation scale. Mothers who had enrolled their children with ID in special schools or receiving services at rehabilitation centres were invited to participate in this study. The data were subjected to the following analyses: mean computation, multivariate analysis of variance, hierarchical regression, and moderation analysis.

**Results:**

The results showed high fatherly support, participation in training, and contribution to the development of their children with ID. However, the mothers’ ratings showed the fathers’ ambivalence toward parenting children with ID. A relationship was found between attitude and support, as well as marital status and the educational level of mothers, providing insight into the involvement of fathers.

**Conclusion:**

The study recommends training programs aimed at improving the attitudes of fathers toward raising children with ID and other study implications.

## Introduction

Intellectual disability (ID) refers to problems associated with mental abilities of individuals which affect two major developmental areas: participation in cognitive activities and adaptive skills [1, 2]. ID is typically diagnosed before the age of 18, and individuals often experience challenges in various developmental areas, including language, communication, social skills, academic activities, and daily living skills [1, 2]. Consequently, individuals with ID are unable to take part in daily living activities in the community [1]. Globally, it has been estimated that 1% of the population is living with a form of ID [1]. In countries such as the United Arab Emirates (UAE), although there is no information about the proportion of the population living with ID, available data show that a little over 2,000 children with ID are enrolled in schools throughout the country [3]. It is useful to mention that ID is widely acknowledged as a lifelong condition, which means that the condition can only be improved with early identification and ongoing support [1]. Children diagnosed with ID need substantial support at home, which facilitates their participation in communal activities, such as education [4–6]. Therefore, both parents are expected to play a formidable role in nurturing and preparing children with ID for community entry and participation in essential services.

Families are comprised of emotionally connected units whose interactions impact the development of their members [7]. This proposition drives scholarly efforts toward understanding family experiences, functioning, or relationships in the event of having children with ID. For the past four decades, there has been interest in exploring the experiences of families raising children with ID [6, 8–13]. Consequently, it has been suggested that the involvement of fathers in the raising of children with ID is believed to have a positive impact on child development [9, 14–16]. In this study, involvement was conceptualized as fathers’ affection, relationships, and action or initiative to acquire knowledge or information to support the development of their children [8, 16]. To this end, involvement is a product of both affective (experiences, attitudes, resilience, challenges, coping strategies, etc.) and behavioral (training and support provided) attitudes toward children with ID.

Significant attention has been paid to the experiences of mothers, and fathers’ perspectives have gained some scholarly attention [9, 10, 14, 17–20]. While studies drawing on only fathers are scarce in non-Western contexts, such as the UAE, available studies have been limited mainly to the affective involvement of fathers in raising children with ID. For instance, studies have reported both positive [10, 21] and negative experiences of fathers [8, 9, 12, 14, 17, 20, 22–25]. These positive experiences include the fathers’ personal growth and bringing the family together to discuss matters concerning the welfare of their children with ID. Conversely, the negative experiences are enormous: concern for the future, limited access to services, stress, and behavior management [14, 25]. Additionally, faith, hope, and motivation are important coping mechanisms that enable fathers to embrace their children with ID [11, 14, 18–20].

Non-Western contexts are the next frontiers in the reformation or development of policies to create conducive environments for the development of children with ID. For instance, in the UAE, there have been some efforts to develop disability laws and policies to enhance inclusion in society [26, 27]. Unfortunately, progress has been slow due to myriad challenges, such as negative attitudes, cultural stereotypes, and the non-enforcement of policies intended for children with ID and their families [28–30]. However, as a cultural society, mothers are dominant when it comes to supporting children with disabilities [31–34], and as such, there is a need for them to evaluate the extent of involvement of their spouses. This research is essential for developing an understanding of the quality of interaction between mothers and fathers in raising children with ID. To extend previous studies, the following research questions were answered:

1. What is the extent of fathers’ involvement in raising children with ID in the UAE?
2. Which mother/child-related factors will impact fathers’ involvement in raising children with ID in the UAE?
3. Which mother/child-related factors predict the involvement of fathers in the raising of children with ID in the UAE?

## Methods

### Study participants

The participants of this study were 158 mothers of children with ID in the UAE (see Table 1). These participants were recruited from the seven Emirates of the UAE to develop a deeper and wider understanding of how fathers who have children with ID are involved in their development. The country has seven states (Emirates), namely, Abu Dhabi, Dubai, Fujairah, Ras Al Khaimah, Sharjah, and Umm Al Quwain. The majority of participants were recruited through text message that was sent by the funding institution of this research. This was the largest drive for recruiting mothers and fathers of children with disabilities in Abu Dhabi. Moreover, Zahed Higher Organization for People of Determination and Ministry of Community Development assisted in sharing the survey with parents.

**Table 1:** Summary of demographic characteristics of participants.

Category (N = 158)	Frequency	Percentage (%)
**Age (n = 157)**		
21–30 years	11	7%
31–40 years	62	40%
41 years and above	84	53%
**Marital status**		
Married	146	92%
Single	12	8%
**Nationality**		
Citizens	120	76%
Residents	38	24%
**Location (n = 157)**		
Abu Dhabi	78	50%
Northern Emirates	79	50%
**Education level**		
Secondary and below	88	56%
Bachelor or above	70	44%
**Employment status**		
Unemployed	106	67%
Employed	52	33%
**Monthly family income**		
Less than AED 10,000	77	49%
AED10,000 –AED20,000	41	26%
AED 21,000 or above	40	25%
**Number of children with ID (n = 94)**		
One child with ID	82	87%
At least two	12	13%
**Years of marriage (n = 142)**		
1–10 years	96	68%
11–20 years	30	21%
21 years or above	16	11%
**Age of children with ID (n = 155)**		
1–12 years	113	73%
13–17 years	42	27%
**Gender of children with ID**		
Male	87	55%
Female	71	45%
**Severity of disability**		
Mild	59	37%
Moderate	83	53%
Severe	16	10%
**Support needs (n = 110)**		
Minimal support	12	11%
Moderate support	46	42%
Substantial support	52	47%
**School enrolment (n = 110)**		
Yes	47	43%
No	63	57%

The inclusion of participants for this study was guided by the following criteria: a) the participant should be the mother of a child with ID; b) the child should be either enrolled in a special school or he or she should be accessing services in a rehabilitation center; c) the child is diagnosed with ID; and d) the mother should be able to provide voluntary consent to participate in this study (see Table 1 for details).

### Instrument

A two-part instrument was used to collect data from the mothers. The section of the instrument regarded the background of the participants (Table 1).

The second part of the instrument included 59 items anchored on a five-point Likert-type scale (1 = strongly disagree and 5 = strongly agree), called the Fathers’ Involvement in Development and Rehabilitation Scale (FIDRS). The instrument was developed to assess mothers’ opinions on fathers’ involvement in caring for and supporting children with ID. The instrument had three domains—support, attitude, and participation in training. The first domain, the support domain, had 37 items. The second domain, attitude toward parenting, included 15 items. The third domain, participation in training, included seven items. Further, the support domain, with 37 items, consisted of three subdomains: personal support, child well-being, and learning and development. Attitude has two subdomains: beliefs toward support and parenting of children with ID. The third domain, training, was a unidimensional scale.

The development of the research instrument was based on an extensive review of the relevant literature. The literature on the different domains helped in writing the items in the questionnaire. A composite mean score from the items in each domain was computed and used to interpret the degree of fathers’ involvement in raising and supporting children with ID.

The first draft of the instrument included 70 raw items. Then, it was examined by three experts for suitability, ease of understanding, orientation to the research problem, and language aspect to maintain the face validity of the instrument. Their feedback was helpful in trimming the items down to 59, and it was piloted before the final implementation.

The reliability of the scale was as follows: support domain, .95 (personal support, .88; learning and development, .95; well-being and development, .95); attitude toward parenting, .92 (belief toward parenting, .78; and beliefs toward support,.90); and participating in rehabilitation and training, .99.

### Procedure

First, the researchers sought approval for the study from the Social Science Research Ethics Committee at UAE University (ERSC_2023_2467). After approval from the committee, the researchers applied for further approval from the Emirates Schools Establishment, Zayed Higher Organization for People of Determination, and Ministry of Community Development to collect data from rehabilitation centers and the Ministry of Education in Abu Dhabi. Before data collection, permission was sought from the Ministry of Education for the Northern Emirates. After receiving these approvals, all special schools in Abu Dhabi and the Northern Emirates were invited to participate in the study. In Abu Dhabi, the Abu Dhabi Early Childhood Authority shared the instrument with parents raising children with disabilities in the Emirate. All the detailed information about the study, with online links to the questionnaire, was forwarded to the potential participants (mothers). They were informed about the purpose of the study, their right to participate or quit the study at any time without any consequences, anonymity of the questionnaire, personal safety of each participant, and voluntary participation.

The data collection was done virtually using an online application called QuestionPro. The instrument was in Arabic and English so that the participants could complete it in their preferred language. Data were collected from February to June 2023. As a motivation for the participants, they were informed that five participants would be randomly chosen for gift cards as an appreciation for their participation. Altogether, 158 mothers signed a written consent form before participating in the study.

### Data analysis

First, the data collected from QuestionPro were imported to Microsoft Excel for cleaning. The data were transferred to IBM SPSS version 29 for further analysis and interpretation. The data were subjected to tests of outliers and normality using boxplots, Q-Q plots, and histograms [35]. The distribution of the data for each domain and subdomain was normally distributed. Based on these results, parametric tests were applied for analysis and interpretation.

Composite mean scores for each domain and subdomain were computed to answer the first research question. For research question 2, a multivariate analysis of variance (MANOVA) was used to examine whether there were any statistically significant differences between participants based on the three aggregate dependent variables (fathers’ support, attitude toward children with ID, support and participation in training). The assumptions of potential outliers, linearity in the distribution of continuous dependent variables, and homogeneity of variances across the test groups were all examined and affirmed [35]. The Bonferroni-adjusted alpha level of .02 was applied as the baseline for examining the significant differences between the research participants. The differences between groups of participants (if significant) were assessed with effect size (partial eta squared), with an interpretation of small within the range of .01–.05), moderate within the range of.06–.1, and large within .1 or above [35].

Afterwards, research question 3 was answered. Initially, Pearson’s correlation was used to examine the association between fathers’ support, attitudes, and participation in training. The degree of correlation was interpreted as follows: small within the range of .10–.30, moderate within the range of.31–.50, and large with .51 or above [35]. A hierarchical regression analysis was applied to examine the impact of fathers’ attitudes and participation in training on their support for children with ID. The regression analysis followed attitude and participation in training in Step 1 to examine their impacts on support without moderation of demographic variables. Then, demographic variables, such as mothers’ age, marital status, nationality, etc., were entered into Step 2 to examine if these variables interfere with the variables in Step 1. The assumptions of normality, linearity, multicollinearity, and homoscedasticity were considered and affirmed [35]. Then, the demographic variables that had a significant association with support were operationalized as moderators to examine their effect on the relationship between the independent and dependent variables [36].

## Results

The mean scores for the scale were as follows: support domain, M = 4.23, SD = .75; (personal support, M = 4.19, SD = .74; learning and development, M = 4.12, SD = .77; and well-being and development, M = 4.10, SD = .83); attitude toward parenting, M = 3.97, SD = .76 (belief toward parenting, M = 4.02, SD = .73; and beliefs toward support, M = 3.93, SD = .85), and participating in rehabilitation and training, M = 4.35, SD = .42.

### Differences between participants

MANOVA was computed to explore the differences between the participants in the dependent variables (see Table 2).

**Table 2:** Difference between participants.

	Wilks’ Lambda	MAN. F	ANOVA F		
			Support	Attitude	Training
**Age**	.95	1.33	2.59	3.65	.99
Effect size		.03	.03	.05	.001
**Marital status**	.70	22.29**	64.74**	34.54**	.69
Effect size		.30	.30	.18	.004
**Nationality**	.99	.16	.44	.15	.006
Effect size		.003	.003	.001	.001
**Location**	.99	.55	.43	.05	.33
Effect size		.01	.003	.001	.006
**Education level**	.92	4.44**	.79	2.14	.005
Effect size		.08	.005	.01	.001
**Employment status**	.97	1.53	.55	.02	2.79
Effect size		.03	.004	.001	.02
**Monthly family income**	.98	.64	.36	.16	.17
Effect size		.01	.005	.002	.002
**Number of children with ID**	.96	1.19	.69	.12	.17
Effect size		.04	.007	.001	.002
**Years of marriage**	.97	.60	.77	.10	.82
Effect size		.01	.01	.001	.01
**Age of children with ID**	.98	.55	.24	.72	.76
Effect size		.01	.003	.009	.01
**Gender of children with ID**	.99	.43	.75	.57	.62
Effect size		.008	.005	.004	.004
**Severity of ID**	.95	1.43	3.51	1.13	.53
Effect size		.03	.04	.01	.007
**Support needs**	.86	2.82**	2.58	.15	2.18
Effect size		.08	.05	.003	.04
**School enrolment**	.96	1.54	3.95	4.22	.66
Effect size		.04	.04	.04	.002

**p < .01

First, a difference was found between participants in marital status on the combined dependent variables, F (3, 153) = 22.29, Wilks’ Lambda = .70, p = .001, with a large effect size, partial eta squared = .30. Individually, a difference was found between participants in the support domain (F [1, 155] = 64.74, p = .001, with a large effect size, partial eta squared = .30) and attitudes toward parenting (F [1, 155] = 34.54, p = .001, with a large effect size, partial eta squared = .18). Regarding support, observation of the mean scores showed that those who were married (M = 4.35, SD = .51) were higher scores than those who had been divorced or widowed (M = 2.82, SD = 1.46). Similarly, those who were married (M = 4.06, SD = .60) indicated a higher fatherly attitude than those who indicated they were single (M = 2.84, SD = 1.41).

Moreover, a difference was found between participants on the combined dependent variable on educational qualification (F [3, 153] = 4.44, Wilks’ Lambda = .92, with a large effect size, partial eta squared = .08) and support needs of children (F [3, 105] = 2.82, Wilks’ Lambda = .86, p = .01, large effect size, partial eta squared = .08). However, individually, no difference was found between the participants in the dependent variables.

### Predictors of paternal involvement

The relationship between the three domains was computed using Pearson moment correlation co-efficient: support to children with ID and attitude (r = .78, p = .001), support to children with ID and rehabilitation and training (r = .06, p = .49), and attitude toward parenting and rehabilitation and training (r = .08, p = .33).

Hierarchical regression was calculated to explore the contribution of attitude and training to the variance in support for children with ID (see Table 3). In Step 1, attitude, and training made a significant contribution to the variance in fatherly support for children with ID (F [2, 91] = 75.54, p = .001). They both contributed 62% of the variance in support for children with ID. However, individually, only attitudes toward parenting children with ID (b = .78, p = .001) significantly contributed to the variance in support for children with ID.

**Table 3:** Attitude and training regressed on fathers’ support to children with ID.

	Uns. Beta	Stand. Error	Stand. Beta	*t*	*p*
**Step 1**					
Attitude towards training	2.02	.17	.78	11.98	.001**
Rehabilitation and training	.40	.90	.03	.44	.66
**Step 2**					
Attitude towards training	1.67	.19	.65	8.90	.001**
Rehabilitation and training	.18	.89	.01	.21	.84
Age	-3.27	4.00	-.06	-.82	.417
Marital status	-27.20	7.21	-.25	-3.77	.001**
Nationality	-3.26	6.19	-.04	-.526	.60
Location	4.04	5.50	.06	-.57	.47
Education level	9.85	4.75	.14	2.07	.04*
Employment status	2.32	5.54	.03	.42	.68
Monthly family income	.10	3.39	.002	.03	.98
Number of children with ID	2.09	6.45	.02	.32	.75
Years of marriage	-1.23	2.80	-.03	-.44	.66
Age of children with ID	3.65	3.48	.072	1.05	.30
Gender of children with ID	-.44	4.36	-.007	-.10	.92
Severity of ID	-5.71	4.17	-.103	-1.37	.18
Support needs	-8.5	3.70	-.17	-2.29	.03*
School enrolment	-2.69	4.68	-.04	-.57	.57

**p ≤ .01

*p ≤ .05

In Step 2, demographic variables were added to the model. The demographic variables made a 12% contribution in the variance in support for children with ID (F [13, 78] = 2.84, p = .002). The combination of demographic variables and independent variables made a 75% significant contribution in the variance in support for children with ID (F [15, 93] = 15.17, p = .001). Individually, four predictors made a significant contribution in support for children with ID: attitude toward parenting children with ID (beta = .65, p = .001), marital status (beta = -.25, p = .001), education level (beta = .13, p = .05), and support needs (beta = -.18, p = .02). Overall, attitude toward parenting children with ID made the most significant contribution to the variance in support to children with ID.

### Moderators of the relationship between attitude and support

Following the hierarchical regression, three demographic variables (marital status, education level, and support needs) were significant in the variance in support needs. Considering this, they were operationalized as moderators.

**Fig 1:**
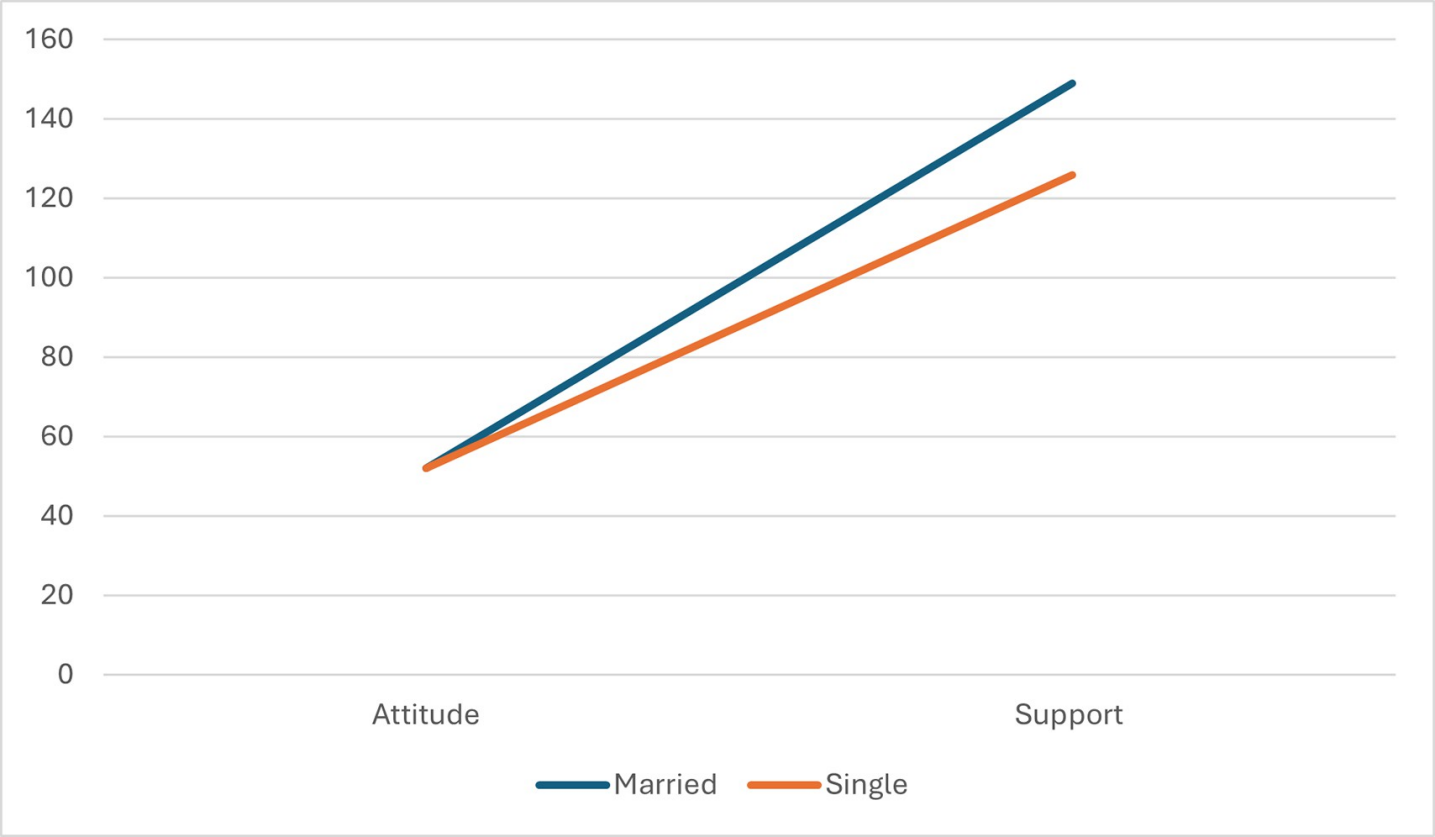
Moderation effect of marital status on attitude and support to children with ID.

First, marital status moderated the relationship between attitude and support for children with ID, beta = .92, 95% CI (.40, 1.45), t = 3.47, p = .001. Individually, participants who were either single (beta = 1.34, 95% CI [1.06, 1.64], t = 9.27, p = .001) or married (beta = 2.27, 95% CI [1.83, 2.71], t = 10.13, p = .001), and a significant relationship was found between attitude and support for children with ID. As shown in Fig 1, married and single participants did not differ in attitude. However, regarding support, married participants indicated more support from fathers than from those who were single.

**Fig 2:**
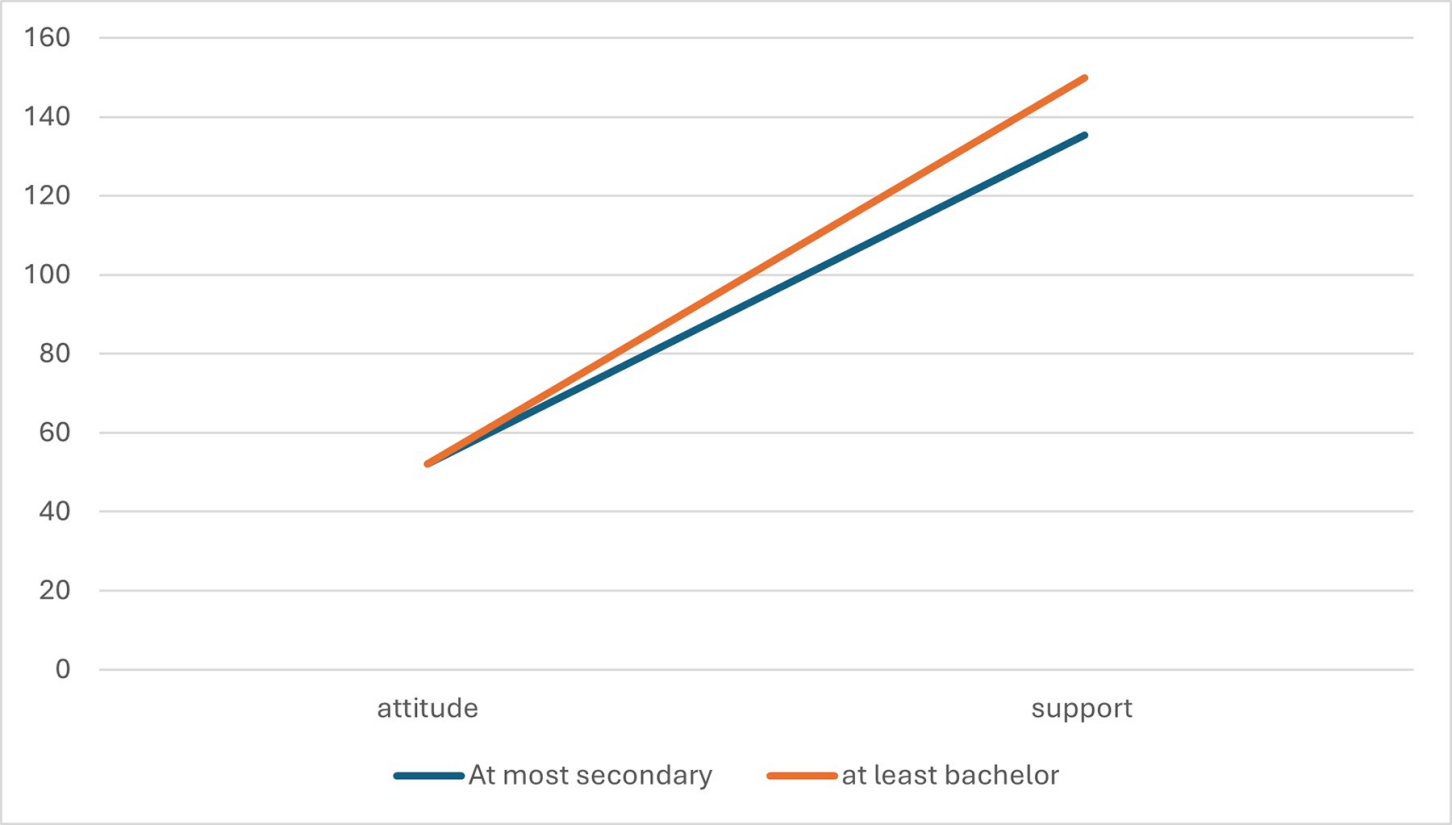
Educational level as moderator of attitude and support to children with ID.

Second, educational level moderated the relationship between attitude and support for children with ID, beta = -.76, 95% CI (-1.23, -.30), t = -3.24, p = .002. Individually, participants either had at most secondary qualifications (beta = 2.24, 95% CI [1.94, 2.53], t = 14.94, p = .001) or at least bachelor degree (beta = 1.47, 95% CI [1.11, 1.83], t = 8.07, p = .001), and a significant relationship was found between attitude and support for children with ID. As shown in Fig 2, participants with secondary and bachelor qualifications did not differ in attitude. However, regarding support, mothers with at least bachelor degree indicated more support from fathers than those who indicated they had at most secondary qualifications. Finally, the level of support required by the children with ID had no interactive effect on the relationship between attitude and support for children with ID, beta = .34, 95% (-.10, .77), t = 1.54, p = .13.

## Discussion

The study presents mothers’ ratings of fathers’ involvement in raising children with ID. The study was conducted against the backdrop of scarce literature on fathers’ involvement in raising children with ID in a non-Western society, such as the UAE. The results showed an overall high level of fathers’ support and participation in training, and contribution to the development of their children with ID. However, mothers’ ratings on the fathers’ attitudes showed the fathers’ perceived uncertainty toward the parenting of a child with ID. Previous studies have reported that fathers may be involved in the development of children with ID; however, they harbor some concerns about raising children with ID [8–10]. The trend identified in this study may not be surprising due to the cultural understanding of disability in UAE society [28–30], which has impacted the attitudes of fathers. It is possible that fathers may be more concerned about societal attitudes towards their children with ID instead of embracing them as equal members of the family and committing to their development in society. This calls for stakeholder engagement between policymakers and fathers to educate on ID, support needs, and the importance of their role in nurturing their children in the UAE.

The study findings also showed a relationship between attitudes and support for raising children with ID. The computation of correlation and hierarchical regression showed the apparent contribution of attitude to the variance in support for raising children with ID. Changing attitudes have been described as the first step toward promoting the acceptance of children with ID in society [37]. Over the years, there have been discussions regarding the difficulties faced by mothers when it comes to raising children with ID [12, 13, 24]. It seemed that tackling fathers’ attitudes toward parenting children with ID could benefit the children. In the literature, some fathers have indicated that they lack knowledge as well as ways to parent children with ID [20]. The findings could provide a useful guide for policymakers, especially in the UAE context. It is apparent that to get fathers actively involved in raising their children, deliberate attempts could be made to change their attitudes and equip them with parenting practices.

Two demographic variables, such as marital status, provided additional insight into the involvement of fathers in raising children with ID. The results of the MANOVA, hierarchical regression, and moderation showed that mothers who were married appeared to receive more support than those who indicated that they were single. In the literature, a shared parenting role between mothers and fathers in the raising of children with ID has been reported [19]. This suggests that fathers who stay with their spouses can offer assistance in terms of teaching self-care or community participation. This seemed not to be the case for mothers who were single and, as such, unable to share responsibilities with their spouses. Indeed, it has been reported that mothers raising children with ID are at high risk of developing mental health problems [31]. This underscores the need for policymakers to develop support programs, such as home visitation by social workers, for mothers who are single and do not receive any support from their spouses.

Second, the educational qualifications of mothers seemed to offer some explanation for the support they received from their spouses. It appears that the higher the educational qualifications of mothers, the more support they receive from their spouses. This finding is somewhat inconsistent with a previous study that reported the limited impact of parents’ educational qualifications in the raising of children with ID [14]. In this study, it may be that mothers who are educated are able to negotiate with spouses regarding the division of labor and areas in which they require their assistance with their children with ID. There is a widely accepted connection between education and access to jobs [38]. Considering this, mothers can share caregiving responsibilities with their spouses. It is fair to postulate that mothers who are less educated may shoulder all caregiving responsibilities. However, conclusions cannot be drawn from this study, and future studies may examine this further.

### Study limitations

This study has several limitations. First, the study included only participants who had children with ID enrolled in either special schools or rehabilitation centers. In this sense, the findings of this study have limited generalizability, even in the UAE. Second, the study examined the role of fathers based on the opinions of mothers, which might have caused response bias, or mothers’ responses may not accurately reflect fathers’ intentions, behaviors, and attitudes. Moreover, it was not possible to verify the mothers’ claims about fathers’ attitudes, support, and participation in training related to raising children with ID. However, the mothers completed the questionnaire in their preferred language, and as such, they might have provided a correct assessment of their spouses’ role in caring for and raising children with ID in the families. Nevertheless, a future study in this area could use fathers’ first-person accounts to comprehend their participation in raising children with ID.

## Conclusion and policy implications

The purpose of this study was to explore, from the mothers’ perspectives, fathers’ involvement in raising children with ID. The findings showed that fathers provided support and participated in training programs to help their children with ID. However, mothers’ ratings noted ambivalence regarding fathers’ attitudes toward parenting children with ID. Also, there was a relationship between attitude and support for children with ID. Additionally, the marital status and educational level of mothers provided additional insight into fathers’ involvement in raising their children with ID.

The UAE government has an undeniable commitment to creating a conducive and inclusive environment for the development of all persons [26, 27]. The study has provided baseline information about the extent of fathers’ involvement in the raising of children with ID and could be considered in future policymaking in the UAE. For instance, policymakers may consider developing training programs aimed at changing fathers’ attitudes toward parenting children with ID. The training program could encompass the etiology, needs, weaknesses, capabilities, strengths of children with ID, and best practices in raising them. Other training programs could target single mothers and those with less education in the UAE. Home visitation programs, as well as fathers’ engagement, could be prioritized to enable fathers to understand their role and ways of supporting the development of children with ID in the UAE.

## Supporting information

S1 File Fathers’ involvement in development and rehabilitation scale (Mohamed et al, 2024). (DOCX)
