# Supplementary material for: Fathers’ involvement in raising children with intellectual disabilities: Mothers’ ratings of the contribution of their spouses
Source: PLoS One. 2024 May 22;19(5):e0294077. doi: 10.1371/journal.pone.0294077 (PMC11111066; doi:10.1371/journal.pone.0294077)
Supplement: S1 File — (DOCX) [file pone.0294077.s001.docx]

***1 = strongly disagree; 2 =disagree; 3= neutral; 4 = agree; 5 = disagree***

**Section A: support**

|  | 1 | 2 | 3 | 4 | 5 |
| --- | --- | --- | --- | --- | --- |
| 1. He shares the responsibility of raising our child of ID. | ❏ | ❏ | ❏ | ❏ | ❏ |
| 1. He is aware of our child's mental and cognitive skills. | ❏ | ❏ | ❏ | ❏ | ❏ |
| 1. He is supportive to our child’s mental and cognitive skills. | ❏ | ❏ | ❏ | ❏ | ❏ |
| 1. He respects the individuality and privacy of our child. | ❏ | ❏ | ❏ | ❏ | ❏ |
| 1. He is aware of how to take care of our child with ID. | ❏ | ❏ | ❏ | ❏ | ❏ |
| 1. He dresses our child or helps him/her to get dressed. | ❏ | ❏ | ❏ | ❏ | ❏ |
| 1. He eats meals with our child with ID. | ❏ | ❏ | ❏ | ❏ | ❏ |
| 1. He participates in providing financial support for our child, such as money and housing. | ❏ | ❏ | ❏ | ❏ | ❏ |
| 1. He participates in providing basic needs for our child, such as food, drinks, and clothing, etc. | ❏ | ❏ | ❏ | ❏ | ❏ |
| 1. He has a good relationship with our child with ID. | ❏ | ❏ | ❏ | ❏ | ❏ |
| 1. He interacts with our child with ID with warmth and love. | ❏ | ❏ | ❏ | ❏ | ❏ |
| 1. He hugs and kisses our child with ID. | ❏ | ❏ | ❏ | ❏ | ❏ |
| 1. He laughs with our child with ID. | ❏ | ❏ | ❏ | ❏ | ❏ |
| 1. He comforts our child with ID when he/she is upset or crying. | ❏ | ❏ | ❏ | ❏ | ❏ |
| 1. He tells our child with ID that he loves them. | ❏ | ❏ | ❏ | ❏ | ❏ |
| 1. Our child with ID feels comfortable and at ease around him. | ❏ | ❏ | ❏ | ❏ | ❏ |
| 1. He talks to/chats with our child with ID | ❏ | ❏ | ❏ | ❏ | ❏ |
| 1. He is accepting of any kind of conversation with our child, no matter what the subject is. | ❏ | ❏ | ❏ | ❏ | ❏ |
| 1. He supports in teaching and guiding our child on what is right and wrong. | ❏ | ❏ | ❏ | ❏ | ❏ |
| 1. He helps our child with ID learn new things and develop their skills. | ❏ | ❏ | ❏ | ❏ | ❏ |
| 1. He assists our child with ID with homework, lessons, and tasks. | ❏ | ❏ | ❏ | ❏ | ❏ |
| 1. He supports our child with ID and assists them in difficult situations. | ❏ | ❏ | ❏ | ❏ | ❏ |
| 1. He teaches religious principles to our child with ID (For example teaching prayer and memorizing the surahs of the Holy Quran). | ❏ | ❏ | ❏ | ❏ | ❏ |
| 1. Our child with ID trusts that their father is a source of healthy and positive support when dealing with challenges and crises. | ❏ | ❏ | ❏ | ❏ | ❏ |
| 1. He is concerned about the mental health of our child with ID. | ❏ | ❏ | ❏ | ❏ | ❏ |
| 1. He is concerned about the physical health of our child with ID. | ❏ | ❏ | ❏ | ❏ | ❏ |
| 1. He is aware of our child's ambitions. | ❏ | ❏ | ❏ | ❏ | ❏ |
| 1. He makes an effort to support the development of our child with ID | ❏ | ❏ | ❏ | ❏ | ❏ |
| 1. He recognizes the skills of our child with ID. | ❏ | ❏ | ❏ | ❏ | ❏ |
| 1. He encourages and praises our child with ID | ❏ | ❏ | ❏ | ❏ | ❏ |
| 1. He accepts the actions and behaviors of our child with ID. | ❏ | ❏ | ❏ | ❏ | ❏ |
| 1. He is aware of the interests of our child with ID | ❏ | ❏ | ❏ | ❏ | ❏ |
| 1. He takes our child with ID to the shops. | ❏ | ❏ | ❏ | ❏ | ❏ |
| 1. He takes our child with ID to centers/schools/clinics. | ❏ | ❏ | ❏ | ❏ | ❏ |
| 1. He takes our child with ID to fun activities such as the zoo or a sporting event. | ❏ | ❏ | ❏ | ❏ | ❏ |
| 1. He plays with toys or puzzles with our child with ID. | ❏ | ❏ | ❏ | ❏ | ❏ |
| 1. He interacts physically with our child with ID, such as by roughhousing or tickling. | ❏ | ❏ | ❏ | ❏ | ❏ |

**Section B: attitude**

|  | 1 | 2 | 3 | 4 | 5 |
| --- | --- | --- | --- | --- | --- |
| 1. One of the most challenging aspects of his life is being a father of a child with ID | ❏ | ❏ | ❏ | ❏ | ❏ |
| 1. He tries his best to raise our child with ID, and he is keen on continuously learning more skills about the different stages of a child's development. | ❏ | ❏ | ❏ | ❏ | ❏ |
| 1. He realizes that our child with ID needs more support than typical children. | ❏ | ❏ | ❏ | ❏ | ❏ |
| 1. He participates in collaboration and dialogue with me about raising our child with ID. | ❏ | ❏ | ❏ | ❏ | ❏ |
| 1. He puts so much into parenting our child with ID and doesn't have time for himself. | ❏ | ❏ | ❏ | ❏ | ❏ |
| 1. He feels overwhelmed with his responsibilities toward our child with ID. | ❏ | ❏ | ❏ | ❏ | ❏ |
| 1. He believes that I am more friendly and patient towards our child of determination, but I need continuous support and motivation to cope with raising our child with ID. | ❏ | ❏ | ❏ | ❏ | ❏ |
| 1. He coordinates with me regarding how our child with ID should be raised. | ❏ | ❏ | ❏ | ❏ | ❏ |
| 1. He shares with me what is going on in his mind at any time about our child with ID. | ❏ | ❏ | ❏ | ❏ | ❏ |
| 1. His commitment to providing proper care for our child with ID makes him a good father. | ❏ | ❏ | ❏ | ❏ | ❏ |
| 1. His top priority is raising our child with ID. | ❏ | ❏ | ❏ | ❏ | ❏ |
| 1. His involvement in parenting our child with ID doesn't interfere or conflict with my parenting style. | ❏ | ❏ | ❏ | ❏ | ❏ |
| 1. His involvement in raising our child with ID helps facilitate my roles and responsibilities. | ❏ | ❏ | ❏ | ❏ | ❏ |
| 1. He disagrees with me about how long he should interact with our child with ID. | ❏ | ❏ | ❏ | ❏ | ❏ |
| 1. He is satisfied with his involvement as a father of a child with ID. | ❏ | ❏ | ❏ | ❏ | ❏ |

**Section C: training**

|  | 1 | 2 | 3 | 4 | 5 |
| --- | --- | --- | --- | --- | --- |
| 1. Attending evidence-based programs (for example, Applied Behavior Analysis, Picture Exchange Communication System, or TEACCH Program) provided him with helpful information towards raising our child with ID. | ❏ | ❏ | ❏ | ❏ | ❏ |
| 1. Attending evidence-based programs helped him develop and learn communication skills suitable for our child with ID. | ❏ | ❏ | ❏ | ❏ | ❏ |
| 1. Attending evidence-based programs helped him use appropriate strategies to support the development of our child with ID. | ❏ | ❏ | ❏ | ❏ | ❏ |
| 1. Attending evidence-based programs enabled him to overcome challenges of raising our child with ID. | ❏ | ❏ | ❏ | ❏ | ❏ |
| 1. Attending evidence-based programs has increased his ability to meet the needs of our child with ID and enhance his/her capabilities. | ❏ | ❏ | ❏ | ❏ | ❏ |
| 1. Attending evidence-based programs contributed to increasing his participation with me in raising our child with ID | ❏ | ❏ | ❏ | ❏ | ❏ |
| 1. Attending an evidence-based training program helped him add new knowledge that enhanced his ability to raise our child with ID | ❏ | ❏ | ❏ | ❏ | ❏ |
